# Isolation and Characterization of *Capnocytophaga bilenii* sp. nov., a Novel *Capnocytophaga* Species Detected in a Gingivitis Subject

**DOI:** 10.3390/pathogens10050547

**Published:** 2021-05-01

**Authors:** Angéline Antezack, Manon Boxberger, Bernard La Scola, Virginie Monnet-Corti

**Affiliations:** 1 Ecole de Médecine Dentaire, Faculté des Sciences Médicales et Paramédicales, Aix-Marseille Université, 27 Boulevard Jean Moulin, 13385 Marseille, France; angeline.antezack@univ-amu.fr; 2 Assistance Publique-Hôpitaux de Marseille (AP-HM), Hôpital Timone, Service de Parodontologie, 264, Rue Saint Pierre, 13385 Marseille, France; 3 Institut de Recherche Pour le Développement (IRD), Assistance Publique-Hôpitaux de Marseille (AP-HM), MEPHI, Aix-Marseille Université, 27 Boulevard Jean Moulin, 13005 Marseille, France; manon.boxberger@hotmail.fr (M.B.); bernard.la-scola@univ-amu.fr (B.L.S.); 4 IHU Méditerranée Infection, 19–21 Boulevard Jean Moulin, 13005 Marseille, France

**Keywords:** *Capnocytophaga*, dental plaque, gingivitis, culturomics, sp. nov.

## Abstract

*Capnocytophaga* species are commensal gliding bacteria that are found in human and animal oral microbiota and are involved in several inflammatory diseases, both in immunocompromised and immunocompetent subjects. This study contributes to increased knowledge of this genus by characterizing a novel species isolated from a dental plaque sample in a male with gingivitis. We investigated morphological and chemotaxonomic characteristics using different growth conditions, temperature, and pH. Cellular fatty acid methyl ester (FAME) analysis was employed with gas chromatography/mass spectrometry (GC/MS). Phylogenetic analysis based on 16S rRNA, orthologous average nucleotide identity (OrthoANI), and digital DNA–DNA hybridization (dDDH) relatedness were performed. The Marseille-Q4570^T^ strain was found to be a facultative aerobic, Gram-negative, elongated, round-tipped bacterium that grew at 25–56 °C and tolerated a pH of 5.5 to 8.5 and an NaCl content ranging from 5 to 15 g/L. The most abundant fatty acid was the branched structure 13-methyl-tetradecanoic acid (76%), followed by hexadecanoic acid (6%) and 3-hydroxy-15-methyl-hexadecanoic acid (4%). A 16S rDNA-based similarity analysis showed that the Marseille-Q4570^T^ strain was closely related to *Capnocytophaga leadbetteri* strain AHN8855^T^ (97.24% sequence identity). The OrthoANI and dDDH values between these two strains were, respectively, 76.81% and 25.6%. Therefore, we conclude that the Marseille-Q4570^T^ strain represents a novel species of the genus *Capnocytophaga*, for which the name *Capnocytophaga bilenii* sp. nov. is proposed (=CSUR Q4570).

## 1. Introduction

The genus *Capnocytophaga* (Gr. n. *kapnos*, smoke; N.L. fem. n. *Cytophaga*, a bacterial genus name; N.L. fem. n. *Capnocytophaga*, bacteria requiring carbon dioxide and related to the cytophaga) belongs to the large family *Flavobacteriaceae* and currently counts 10 species with a validly published and correct name [1]. *Capnocytophaga* species are primarily commensals of the oral cavity in humans and animals, especially dogs and cats. They are recognized as opportunistic pathogens, leading to various extra-oral infections, including severe sepsis [2], bloodstream infections [3], abscess [4,5], vertebral osteomyelitis [6], pneumonia [7], and perinatal infections [8] in both immunocompetent and immunosuppressed patients. In addition, *Capnocytophaga* species have been thought to play a role in cancer development. For example, *Capnocytophaga gingivalis* has been identified as strongly correlated with oral squamous cell carcinoma (OSCC) and has been described as a promising diagnostic marker [9,10]. The genus *Capnocytophaga* was also found in increased amounts in the saliva of lung cancer patients [11]. Moreover, several studies have reported members of the genus *Capnocytophaga* as periodontal pathogens [12,13,14]. Periodontal diseases are multifactorial inflammatory pathologies that are characterized by progressive destruction of the tooth-supporting apparatus [15]. An increase in an abundance of *Capnocytophaga* species was found in subjects with gingivitis [16] and periodontitis [17]. Furthermore, the genus *Capnocytophaga* is one of the main sources of β-lactamases in the oral cavity and constitutes the main oral reservoir of macrolide–lincosamide–streptogramin genes, adding to their pathogenicity [18].

In this study, we used the rapid and precise routine identification by matrix-assisted laser desorption ionization time-of-flight (MALDI-TOF) mass spectrometry (MS) for the identification of an unknown strain, which was isolated from a dental plaque sample from a 25-year-old male with gingivitis. The Marseille-Q4570^T^ strain was described using morphological examinations and biochemical characteristics and compared to its closely related phylogenetic neighbors. We propose for this strain the species name *Capnocytophaga bilenii* sp. nov (=CSUR Q4570).

## 2. Results

### 2.1. Strain Identification and Classification

**Figure 1:**
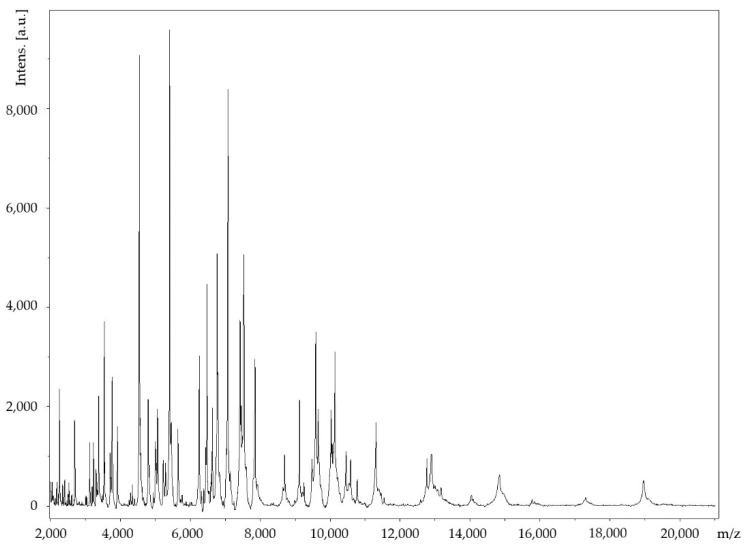
MALDI-TOF MS reference mass spectrum for the Marseille-Q4570^T^ strain. Spectra from 12 individual colonies were compared and a reference spectrum was generated.

The Marseille-Q4570^T^ strain was isolated from a dental plaque sample of a 25-year-old male with gingivitis living in Marseille, France. The Marseille-Q4570^T^ strain could not be identified by MALDI-TOF MS, as the score was lower than 1.8 (Figure 1).

**Figure 2:**
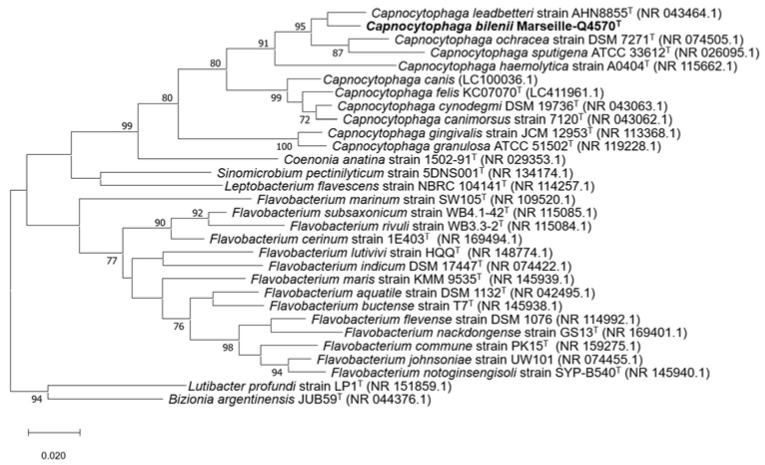
Maximum likelihood tree based on the comparison of 16S rRNA gene sequences showing the phylogenetic relationships of the Marseille-Q4570^T^ strain and other closely related species. Bootstrap values (expressed as percentages of 1000 replications) are displayed at the nodes. Only bootstrap values of 70% or greater are shown. Type strains are indicated with superscript T. GenBank accession numbers of 16S rRNA are indicated in parentheses. Sequences were aligned using MUSCLE (MUltiple Sequence Comparison by Log Expectation) with default parameters, and phylogenetic inference was obtained using the maximum likelihood method and MEGA X software [20]. Bootstrap values obtained by repeating the analysis 1000 times to generate a majority consensus tree are indicated at the nodes. There was a total of 1409 positions in the final dataset.

The 16S rDNA-based similarity analysis of the Marseille-Q4570^T^ strain against GenBank yielded the highest nucleotide sequence similarities of 97.24% sequence identity with *Capnocytophaga leadbetteri* strain AHN8855^T^ (GenBank accession no. NR_043464.1). As this value was lower than the 98.65% threshold for differentiating two species [19], the Marseille-Q4570^T^ strain was considered to be a potential new species within the genus *Capnocytophaga*. The 16S rRNA gene sequence was deposited into GenBank under the accession number MW762958. The phylogenetic tree highlighting the position of the Marseille-Q4570^T^ strain relative to other closely related species is shown in Figure 2.

### 2.2. Phenotypic Characteristics

**Figure 3:**
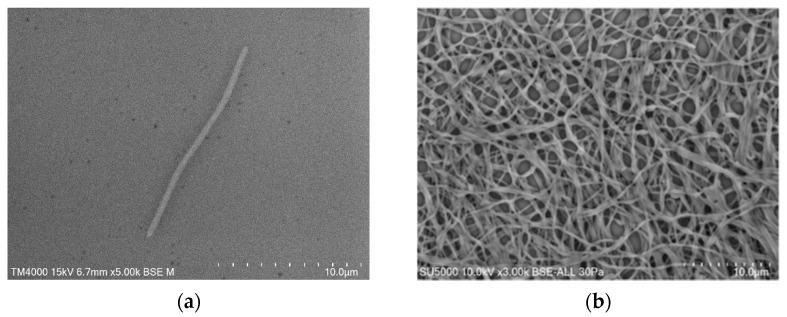
Micrograph electron microscopy of the Marseille-Q4570^T^ strain. (**a**) A single cell after 48 h growth on Columbia agar with 5% sheep blood (TM4000 SEM, Hitachi High-Tech, HHT, Tokyo, Japan), and (**b**) the network organization of the cells (SU5000 FE-SEM, Hitachi High-Tech, HHT, Tokyo, Japan). Scales and acquisition settings are shown in the figure.

Growth was observed on Columbia agar with 5% sheep blood (BioMérieux, Marcy l’Etoile, France) at 37 °C after 48 h of incubation in an aerobic atmosphere. Growth was also achieved in anaerobic (AnaeroGen Compact; Oxoid, Thermo Scientific, Dardilly, France) and microaerophilic atmospheres (campyGEN; Oxoid, Thermo Scientific, Dardilly, France). The temperature range of the strain was determined to be 25–56 °C, with an optimum growth temperature of 37 °C. The bacterial cells tolerated a pH of 5.5 to 8.5 (optimum pH 5.5) and an NaCl content ranging from 5 to 15 g/L (optimum 5 g/L). Colony appearance on Columbia agar with 5% sheep blood (BioMérieux, Marcy l’Etoile, France) incubated at 37 °C for 2 days was yellow-orange, smooth, and shiny. Cells were slender (0.3–0.4 × 8.5–17 μm), elongated, round-tipped, and Gram-negative, as determined by scanning electron microscopy (SEM) (Figure 3a). The cells were intertwined to form a dense network (Figure 3b).

Using an API 50 CH strip, positive results were shown for D-galactose, D-glucose, D-fructose, D-mannose, methyl αD-mannopyranoside, methyl αD-glucopyranoside, N-acetyl-glucosamine, amygdalin, arbutin, esculin ferric citrate, salicin, D-cellobiose, D-maltose, D-lactose, D-melibiose, D-saccharose, D-trehalose, inulin, D-melezitose, D-raffinose, amidon, glycogen, xylitol, gentiobiose, and D-turanose. Using an API ZYM strip, positive reactions were obtained for alkaline phosphatase, C4 esterase, C8 esterase lipase, leucine arylamidase, valine arylamidase, cystine arylamidase, trypsin, α-chymotrypsin, acid phosphatase, naphthol-AS-BI-phosphohydrolase, α-glucosidase, and N-acetyl-β-glucosaminidase. In addition, the Marseille Q4570^T^ strain was negative for oxidase and catalase activity. The comparison of phenotypic characteristics between the Marseille-Q4570^T^ strain and other *Capnocytophaga* species is listed in Table 1.

The most abundant fatty acid was the branched structure 13-methyl-tetradecanoic acid (76%), followed by hexadecanoic acid (6%) and 3-hydroxy-15-methyl-hexadecanoic acid (4%). Several other unsaturated, branched, and specific 3-hydroxy structures were also described. This fatty acid profile corresponds to the commonly described compositions for *Capnocytophaga* strains [30] (Table 2).

### 2.3. Genome Sequencing Information and Genome Properties

**Figure 4:**
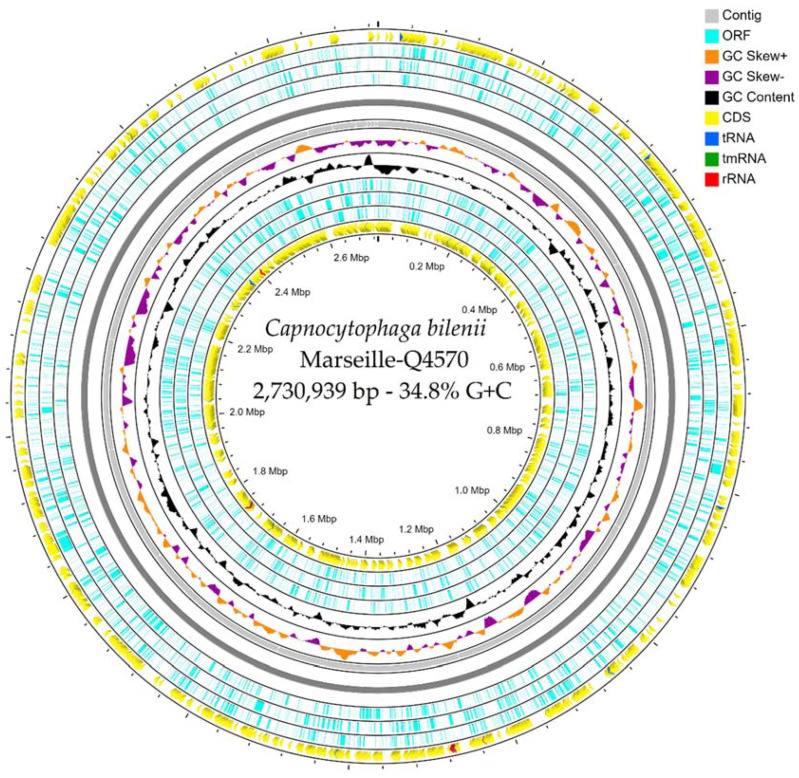
A circular map generated using the CGView Server^BETA^ [31] showing a complete view of the genome of the Marseille-Q4570^T^ strain.

The genome size of the Marseille-Q4570^T^ strain was 2,730,939 bp long with a 38.4% G+C content. It was assembled into 20 contigs with a mean coverage of 31.0%. It was deposited into GenBank under the accession number JAGDYP010000000. Of the 2512 predicted genes, 2460 were protein-coding genes and 52 were RNAs (four 5S rRNA, two 16S rRNA, two 23S rRNA, 41 tRNA, and three ncRNA). There were 961 genes with putative function (by COGs) for the Marseille-Q4570^T^ strain (Table 3). Finally, 1388 genes (55.3%) were annotated as hypothetical proteins for the Marseille-Q4570^T^ strain. A circular map showing a complete view of the genome of the Marseille-Q4570^T^ strain is shown in Figure 4.

### 2.4. Comparison to Closely Related Bacterial Strains

The genome of the Marseille-Q4570^T^ strain was compared to the available genomes of nine closely related bacterial strains: *Capnocytophaga canimorsus*, *C. cynodegmi*, *C. gingivalis*, *C. haemolytica*, *C. leadbetteri*, *C. ochracea*, *C. sputigena*, *Flavobacterium johnsonia*, and *Flavobacterium lutivivi*. The genome size of our strain (2.7 Mb) was larger than that of *C. canimorsus* (2.4 Mb), *C. cynodegmi* (2.6 Mb), *C. haemolytica* (2.6 Mb), and *C. ochracea* (2.6 Mb). In addition, the G+C content of our strain (38.4%) was equal to that of *C. sputigena* and higher than that of *F. lutivivi* (32.4%), *F. johnsonia* (34.1%), *C. cynodegmi* (34.4%), and *C. canimorsus* (36.3%).

**Figure 5:**
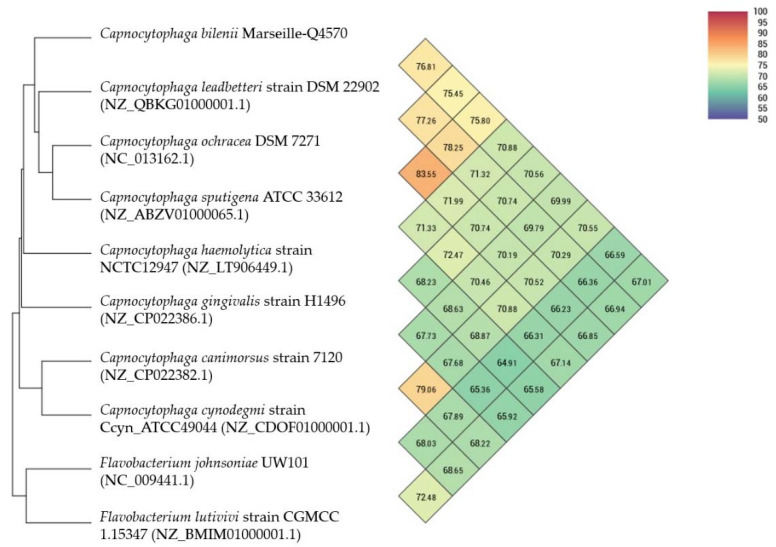
Heatmap generated with orthologous average nucleotide identity (OrthoANI) values calculated using OAT software between the Marseille Q4570^T^ strain and nine other closely related species.

Using dDDH analysis, the Marseille-Q4570^T^ strain exhibited values ranging from 33.5% [31.1–36%] with C. gingivalis to 21.0% [18.7–23.4%] with F. johnsoniae (Table 4). These values are lower than the 70% threshold used for delineating prokaryotic species, thus confirming that the Marseille-Q4570^T^ strain represents a new species [10]. In addition, using OrthoANI analysis, the Marseille-Q4570^T^ strain exhibited values ranging from 76.81% with C. leadbeterri to 67.01% with F. lutivivi (Figure 5).

**Figure 6:**
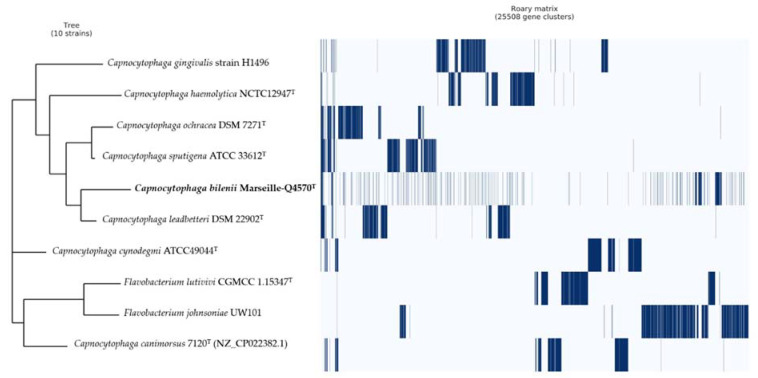
Pangenome analysis of the Marseille-Q4570^T^ strain’s whole-genome sequences. A maximum likelihood tree was constructed from accessory genome elements (left). The presence (blue) and the absence (white) of accessory genome elements are presented on the right.

Pangenome analysis of the Marseille-Q4570^T^ strain showed a total of 25,508 gene clusters distributed as follows: core genes = 2, soft core genes = 0, shell genes = 1037, and cloud genes = 24,469, respectively (Figure 6).

### 2.5. Description of Capnocytophaga bilenii nov. sp.

Capnocytophaga bilenii (bi.le.nii N.L. gen. masc. n. bilenii, from Bilen, named after the French clinical microbiologist Melhem Bilen, an expert in new species isolation).

The cells were Gram-negative, facultative aerobic, elongated round-tipped bacteria, approximately 0.3 to 0.4 μm wide and 8.5 to 17 μm long. Colonies on Columbia agar with 5% sheep blood (BioMérieux, Marcy l’Etoile, France) incubated at 37 °C for 2 days were yellow-orange, smooth, and shiny. The temperature range for growth was 25–56 °C (optimum 37 °C). The bacterial cells tolerated a pH of 5.5 to 8.5 (optimum pH 5.5) and an NaCl content ranging from 5 to 15 g/L (optimum 5 g/L). Using an API 50 CH strip, positive results were shown for D-galactose, D-glucose, D-fructose, D-mannose, methyl αD-mannopyranoside, methyl αD-glucopyranoside, N-acetyl-glucosamine, amygdalin, arbutin, esculin ferric citrate, salicin, D-cellobiose, D-maltose, D-lactose, D-melibiose, D-saccharose, D-trehalose, inulin, D-melezitose, D-raffinose, amidon, glycogen, xylitol, gentiobiose, and D-turanose. According to the API ZYM system, cells were positive for alkaline phosphatase, C4 esterase, C8 esterase lipase, leucine arylamidase, valine arylamidase, cystine arylamidase, trypsin, α-chymotrypsin, acid phosphatase, naphtol-AS-BI-phosphohydrolase, α-glucosidase, and N-acetyl-β-glucosaminidase. The most abundant fatty acid was the branched structure 13-methyl-tetradecanoic acid (76%), followed by hexadecanoic acid (6%) and 3-hydroxy-15-methyl-hexadecanoic acid (4%). The genome size of the Marseille-Q4570^T^ strain was 2.7 Mb long with a 38.4% G+C content. The type strain, Marseille-Q4570^T^ (CSUR Q4570), was isolated from a sample of dental plaque of a male with gingivitis. The sequence data of the Marseille-Q4570^T^ 16S rRNA gene and the whole genome were deposited in the GenBank database under accession numbers MW762958 and JAGDYP010000000, respectively.

## 3. Discussion

In this study, we describe a novel species belonging to the genus *Capnocytophaga* isolated from a dental plaque sample of a male with gingivitis living in Marseille, France. The other *Capnocytophaga* species currently described are also generally isolated from the human oral cavity (*C. gingivalis*, *C. granulosa*, *C. haemolytica*, *C. leadbetteri*, *C. ochracea*, and *C. sputigena*), and also that of dogs and cats (*C. canimorsus*, *C. cynodegmi*, and *C. canis*) [33]. Among members of the genus *Capnocytophaga*, the Marseille-Q4570^T^ strain shared the highest 16S rRNA gene sequence similarities (97.24% sequence identity) with *C. leadbetteri* strain AHN8855^T^, an anaerobic Gram-negative rod bacterium isolated from the oral cavity of children [21]. According to the literature, little is known about *C. leadbetteri* pathogenicity. To our knowledge, only two *C. leadbetteri* infections have been previously reported: chorioamnionitis in a 33-year-old immunocompetent pregnant woman [8] and febrile interstitial lung pneumonia in a 66-year-old HIV-positive male [7]. Recently, the association between *C. leadbetteri* and cancer was investigated. An abundance of *C. leadbetteri* was found to be significantly increased in subjects with OSCC, and the expression of genes involved in bacterial chemotaxis, flagellar assembly, and lipopolysaccharide biosynthesis were shown to be significantly elevated, suggesting a potential association between this microorganism and OSCC [34]. Another study identified *C. leadbetteri* as a potential biomarker of thick white coating in patients with gastric cancer [35]. In an exploratory study, Acharya et al. found that *C. leadbetteri* was significantly more abundant in the saliva of treated and well-maintained chronic periodontitis subjects than in healthy controls with similar bleeding on probing score, suggesting that the salivary microbiome might be used as a biomarker to identify periodontitis-susceptible subjects [17]. These preliminary results are particularly promising, as periodontal disease is at high risk of recurrence [36]. Here, the Marseille-Q4570^T^ strain was isolated from a dental plaque sample of a male with gingivitis, and further studies are needed to investigate the putative association between this strain and gingival inflammation.

The genomic content (dDDH, orthoANI, and pangenome) and biochemical characteristics clearly indicated that the Marseille-Q4570^T^ strain could be differentiated from other *Capnocytophaga* species. Based on the results from phenotypic, chemotaxonomic, genomic, and phylogenetic analyses, we concluded that the Marseille-Q4570^T^ strain represents a novel species of the genus *Capnocytophaga*, for which the name *Capnocytophaga bilenii* sp. nov. is proposed (=CSUR Q4570).

## 4. Materials and Methods

### 4.1. Strain Isolation and Phenotypic Tests

A sample of dental plaque was collected from a 25-year-old male with gingivitis living in Marseille, France. The patient gave informed consent, and the study was approved by the Comité de Protection des Personnes (C.P.P.) Sud-Ouest et Outre-Mer 1 (no. ID RCB: 2020-A01234-35—CPP 1-20- 075 ID 9806). Briefly, the sampling area was first isolated by cotton rolls and gently air dried for 5 s to remove any saliva present. Supra-gingival plaque was collected using a sterile curette and placed in a 1.5 mL Eppendorf tube containing 1.0 mL of Aé-Ana transport medium (Culture-Top, Eurobio scientific, Les Ulis, France). After vortexing for 30 s, a 10-fold dilution series of the sample was prepared in phosphate-buffered saline 1×. Columbia sheep blood agar plates (BioMérieux, Marcy l’Etoile, France) were inoculated with 50 μL each of 10^−4^ to 10^−8^ diluted plaque suspension. After 48 h of incubation in an aerobic atmosphere at 37 °C, the culture plates were inspected using a magnifying glass and any microcolonies or colonies showing satellitism were passaged onto a fresh Columbia agar sheep blood plate. MALDI-TOF MS protein analysis was performed with a Microflex LT mass spectrometer (Bruker Daltonics, Bremen, Germany; external mass spectrometer calibration accuracy ± 300 ppm), as previously reported [19]. The obtained spectra were imported into BioTyper-RTCTM version 3.0 software (Bruker Daltonics GmbH) and analyzed by standard pattern matching (with default parameter settings). Interpretation of the scores was carried out as previously reported [19]. One purified strain, designated Marseille-Q4570^T^ and deposited in the Collection de Souches de l’Unité des Rickettsies under accession number Q4570, could not be identified by MALDI-TOF MS.

Gram staining was carried out using standard Gram stain, and morphological characteristics were observed using a scanning electron microscope (TM4000 SEM, Hitachi High-Tech, HHT, Tokyo, Japan) and a field emission scanning electron microscope (SU5000 FE-SEM, Hitachi High-Tech, HHT, Tokyo, Japan) with cultures grown on Columbia agar with 5% sheep blood (BioMérieux, Marcy l’Etoile, France) at 37 °C for 48 h under aerobic conditions. A colony was collected from agar and immersed in a 2.5% glutaraldehyde fixative solution. The slide was gently washed in water, air-dried, and examined with a TM4000 SEM and a SU5000 FE-SEM operated at 15.0 kV and 10.0 kV, respectively.

Subculture of the Marseille-Q4570^T^ strain was attempted at a wide range of temperatures (25, 28, 31.5, 37, 41.5, and 56 °C) on Columbia agar with 5% sheep blood, and in different conditions of pH (5.5, 6.5, 7.5, and 8.5) and salinity (5, 10, and 15 g/L) on Columbia agar base (bioMérieux, Marcy l’Etoile, France). Growth of the strain was also tested in anaerobic (AnaeroGen Compact; Oxoid, Thermo Scientific, Dardilly, France) and microaerophilic (campyGEN; Oxoid, Thermo Scientific, Dardilly, France) conditions at 37 °C for 48 h. API ZYM and API 50 CH kits (bioMérieux, Marcy l’Etoile, France) were used to perform biochemical analysis according to the manufacturer’s instructions. To evaluate whether our strain was able to form spores, heat shock at 80 °C for 10 min was conducted. Oxidase (MASTDISCS^®^ ID, Mast Group Ltd., Bootle, Merseyside, United Kingdom) and catalase (bioMérieux, Marcy l’Etoile, France) assays were also performed. Finally, fatty acid methyl ester (FAME) analysis by GC/MS cellular fatty acid methyl ester (FAME) analysis was performed by GC/MS. Two samples were prepared with approximately 20 mg of bacterial biomass per tube harvested from several culture plates. Fatty acid methyl esters were prepared as described by Sasser [21]. GC/MS analyses were carried out as previously described [22]. Briefly, fatty acid methyl esters were separated using an Elite 5-MS column and monitored by mass spectrometry (Clarus 500—SQ 8 S, Perkin Elmer, Courtaboeuf, France). A spectral database search was performed using MS Search 2.0 operated with the Standard Reference Database 1A (NIST, Gaithersburg, MD, USA) and the FAME mass spectral database (Wiley, Chichester, UK).

### 4.2. Extraction and Genome Sequencing

Genomic DNA was extracted using the EZ1 biorobot (Qiagen, Courtaboeuf, Les Ulis, France) with the EZ1 DNA tissue kit and then sequenced on the MiSeq technology (Illumina, San Diego, CA, USA) with the Nextera Mate Pair sample prep kit and Nextera XT Paired end (Illumina, San Diego, CA, USA), as previously described [23]. In order to improve the genome sequence, an Oxford Nanopore approach was performed on 1D genomic DNA sequencing for the MinIon device using an SQK-LSK109 kit. The library was constructed from 1 µg genomic DNA without fragmentation and end repair. Adapters were ligated to both ends of genomic DNA. After purification on AMPure XP beads (Beckman Coulter Inc, Fullerton, CA, USA), the library was quantified by a Qubit assay with the high sensitivity kit (Life technologies, Carlsbad, CA, USA). A total of 1047 active pores were detected for the sequencing and the WIMP workflow was chosen for bioinformatic analysis in real time. After 1 h of run time and end life of the flowcell, 617,960 reads were generated as raw data.

### 4.3. Assembly and Annotation of the Genome Sequence

The assembly was performed with a pipeline incorporating different software (Velvet [37], Spades [38], Soap Denovo [39]) and trimmed data (MiSeq and Trimmomatic [40] software) or untrimmed data (only MiSeq software). GapCloser was used to reduce assembly gaps. Scaffolds < 800 bp and scaffolds with a depth value < 25% of the mean depth were removed. The best assembly was selected using different criteria (number of scaffolds, N50, number of N).

Prokka (Galaxy v 1.14.5) was used for prediction in the Open Reading Frame (ORF) with the default settings [41]. Deviations in the sequencing regions predicted by ORFs were excluded. BlastP was used to predict the bacterial proteome (E value of 1e03, coverage of 70%, and percent identity of 30%) according to the Orthological Group (COG) database. In the absence of a match, the search for BlastP in the database [42] was extended with an E value of 1e03, coverage of 70%, and percent identity of 30%. However, if the length of the sequence was less than 80 amino acids (aa), an E value of 1e05 was used. The rRNA and tRNA genes were retrieved using Prokka (Galaxy v 1.14.5) [43,44]. In addition, CGView Server^BETA^ [31] was used to generate a circular map showing a complete view of the genome of the Marseille-Q4570^T^ strain.

### 4.4. Phylogenetic Analysis and Genome Comparison

The 16S rRNA gene sequence of the Marseille-Q4570^T^ strain was obtained and compared to the most closely related species retrieved using NCBI BLAST (National Center for Biotechnology Information, Basic Local Alignment Search Tool; https://blast.ncbi.nlm.nih.gov/Blast.cgi, accessed on 5 January 2021) and then submitted to the GenBank database. Phylogenetic analyses were performed using MEGA X software [23], with genetic distances determined according to the Kimura two-parameter model [24] and phylogenies reconstructed with the maximum-likelihood method. The topology of the phylogenetic tree was conducted using the bootstrap method with 1000 repetitions. All positions containing gaps and missing data were eliminated from the dataset (complete deletion option). To estimate the mean level of nucleotide sequence similarity at the genome level between the Marseille-Q4570^T^ strain and closely related species, the digital DNA–DNA hybridization (dDDH) and the orthologous average nucleotide identity (OrthoANI v 0.93.1) parameters were calculated using the OAT [45] and GGDC (Genome-to-Genome Distance Calculator v 2.1) [32] software programs, respectively. The Pangenome distribution of the Marseille-Q4570^T^ strain and other closely related species was evaluated using Roary software (Galaxy v 3.13.0) [46].

## Figures and Tables

**Table 1:** Phenotypic and biochemical characterization of the Marseille-Q4570^T^ strain compared with other *Capnocytophaga* species.

Characteristics	1	2	3	4	5	6	7	8	9
Oxidase activity	−	−	−	−	−	+	+	−	−
Catalase activity	−	−	−	−	−	+	+	−	−
Fermentation of:									
Amygdalin	w	−	+	w	−	ND	ND	ND	ND
Cellobiose	w	−	+	−	−	+	−	ND	ND
Fructose	+	−	+	−	+	+	−	+	ND
Galactose	+	w	+	+	+	+	+	ND	ND
Glucose	+	w	+	+	+	+	+	+	ND
Lactose	+	w	+	+	+	+	+	ND	ND
Raffinose	+	−	+	+	w	+	−	ND	ND
API ZYM									
Alkaline phosphatase	+	+	+	+	+	+	+	+	+
C_4_ esterase	+	w	−	−	+	+	+	+	+
C_8_ esterase lipase	+	w	+	+	+	+	+	+	+
C_14_ lipase	−	ND	−	ND	−	−	−	−	−
Leucine arylamidase	+	+	+	+	+	+	+	+	+
Valine arylamidase	+	+	+	+	+	+	+	+	+
Cystine arylamidase	+	+	+	−	+	−	−	+	+
Trypsin	+	−	−	+	−	+	+	−	+
α−Chymotrypsin	+	w	−	w	−	−	−	−	+
Acid phosphatase	+	+	+	+	+	+	+	+	+
Naphthol−AS−BI−Phosphohydrolase	+	ND	+	ND	+	+	+	+	+
α−Galactosidase	−	ND	−	ND	−	−	−	−	−
β−Galactosidase	−	+	+	w	+	−	−	+	−
β−Glucuronidase	−	−	−	−	−	−	−	−	−
α−Glucosidase	+	+	+	+	+	+	+	+	+
β−Glucosidase	−	−	−	−	+	−	−	−	+
N−Acetyl−β−Glucosaminidase	+	+	+	+	−	+	+	+	−
α−Mannosidase	−	−	−	−	−	−	−	−	−
α−Fucosidase	−	−	−	−	−	−	+	−	−

API ZYM and API 50 CH test kits (bioMérieux, Marcy l’Etoile, France) were used for the characterization of the Marseille-Q4570^T^ strain. Strains: 1, Marseille-Q4570^T^; 2, *Capnocytophaga leadbetteri* strain AHN8855^T^ [21]; 3, *Capnocytophaga ochracea* [1,21,22]; 4, *Capnocytophaga haemolytica* [21,23]; 5, *Capnocytophaga granulosa* [21,23,24]; 6, *Capnocytophaga cynodegmi* [25,26]; 7, *Capnocytophaga canimorsus* strain 7120^T^ [26,27]; 8, *Capnocytophaga sputigena* [1,28]; 9, *Capnocytophaga gingivalis* [1,29].+, positive; w, weakly positive; −, negative; ND, no data available.

**Table 2:** Cellular fatty acid compositions of the Marseille-Q4570^T^ strain and other *Capnocytophaga* species.

Fatty Acid	1	2	3	4
C_13:0_	TR	ND	ND	ND
C_13:0_ iso	1.3	–	3	TR
C_14:0_	1.8	TR	TR	TR
C_14:0_ iso	TR	ND	ND	ND
C_14:0_ 3-OH	TR	ND	ND	ND
C_15:0_	TR	TR	TR	TR
C_15:0_ iso	75.6	61	75	78
C_15:0_ 3-OH iso	1.7	3	2	3
C_16:0_	5.8	12	3	4
C_16:0_ 3-OH	1.7	2	4	4
C_17:0_	TR	TR	TR	TR
C_17:0_ iso	TR	ND	ND	ND
C_17:0_ 3-OH iso	4.1	2	8	7
C_17:0_ anteiso	TR	ND	ND	ND
C_18:0_	1.3	4	TR	2
C_18:1n9_	2.8	6	2	TR
C_18:2n6_	2.7	10	3	2

All values are given as a percentage of total fatty acids. Strains: 1, Marseille-Q4570^T^; 2, *C. ochracea* 25^T^ [30]; 3, *C. gingivalis* 27^T^ [30]; *C. sputigena* 4^T^ [30]. –, Not detected; TR, trace amounts (<1%); ND, no data available.

**Table 3:** Number of genes associated with the clusters of orthologous group (COG) functional categories of the Marseille-Q4570^T^ strain.

Code	Marseille-Q4570^T^ Strain	Description
[J]	129	Translation, ribosomal structure, and biogenesis
[A]	0	RNA processing and modification
[K]	40	Transcription
[L]	67	Replication, recombination, and repair
[B]	1	Chromatin structure and dynamics
[D]	12	Cell cycle control, cell division, and chromosome partitioning
[Y]	0	Nuclear structure
[V]	21	Defense mechanisms
[T]	15	Signal transduction mechanisms
[M]	74	Cell wall/membrane/envelope biogenesis
[N]	2	Cell motility
[Z]	0	Cytoskeleton
[W]	0	Extracellular structures
[U]	20	Intracellular trafficking, secretion, and vesicular transport
[O]	50	Posttranslational modification, protein turnover, and chaperones
[X]	0	Mobilome: prophages, transposons
[C]	63	Energy production and conversion
[G]	46	Carbohydrate transport and metabolism
[E]	87	Amino acid transport and metabolism
[F]	47	Nucleotide transport and metabolism
[H]	65	Coenzyme transport and metabolism
[I]	34	Lipid transport and metabolism
[P]	48	Inorganic ion transport and metabolism
[Q]	20	Secondary metabolites biosynthesis, transport, and catabolism
[R]	118	General function prediction only
[S]	69	Function unknown

**Table 4:** Numerical DNA–DNA hybridization values (%) obtained by comparison between the Marseille-Q4570^T^ strain and other closely related species using the Genome-to-Genome Distance Calculator 2 (GGDC 2) [32]. The confidence intervals indicate the inherent uncertainty in estimating DNA–DNA hybridization values from intergenomic distances based on models derived from empirical test data sets.

Species	1	2	3	4	5	6	7	8	9	10
1 *Capnocytophaga bilenii* Marseille-Q4570^T^	100.00	33.5 (31.1–36)	25.6 (23.3–28.1)	25.6 (23.3–28.1)	24.0 (21.7–26.5)	23.7 (21.4–26.1)	23.4 (21.1–25.8)	22.5 (20.2–25)	22.1 (19.9–24.6)	21.0 (18.7–23.4)
2 *Capnocytophaga gingivalis*		100.00	32.4 (30–34.9)	35.2 (32.7–37.7)	20.0 (17.8–22.4)	30.4 (28–32.9)	23.4 (21.1–25.9)	23.5 (21.2–25.9)	28.0 (25.7–30.5)	22.9 (20.6–25.4)
3 *Capnocytophaga leadbetteri*			100.00	24.8 (22.5–27.3)	25.4 (23.1–27.9)	23.7 (21.4–26.1)	22.0 (19.7–24.4)	23.7 (21.4–26.2)	21.5 (19.3–24)	23.5 (21.2–25.9)
4 *Capnocytophaga sputigena*				100.00	23.3 (21–25.7)	31.0 (28.6–33.5)	20.0 (17.8–22.4)	22.0 (19.7–24.4)	22.2 (19.9–24.7)	26.6 (24.3–29.1)
5 *Flavobacterium lutivivi*					100.00	22.0 (19.7–24.4)	19.1 (16.9–21.5)	21.6 (19.3–24)	27.8 (25.4–30.3)	19.1 (17–21.5)
6 *Capnocytophaga ochracea*						100.00	23.3 (21–25.8)	23.4 (21.1–25.9)	26.6 (24.3–29.1)	30.1 (27.8–32.7)
7 *Capnocytophaga cynodegmi*							100.00	44.2 (41.7–46.8)	22.7 (20.4–25.1)	24.5 (22.2–27)
8 *Capnocytophaga canimorsus*								100.00	24.5 (22.2–27)	21.2 (18.9–23.6)
9 *Capnocytophaga haemolytica*									100.00	29.0 (26.6–31.5)
10 *Flavobacterium johnsoniae*										100.00

## Data Availability

The data presented in this study are contained within the article.
